# The impact of indoor air temperature on the executive functions of human brain and the physiological responses of body

**DOI:** 10.15171/hpp.2019.07

**Published:** 2019-01-23

**Authors:** Ali Mohammad Abbasi, Majid Motamedzade, Mohsen Aliabadi, Rostam Golmohammadi, Leili Tapak

**Affiliations:** ^1^Department of Occupational Hygiene, Research Center for Health Sciences, Hamadan University of Medical Sciences, Hamadan, Iran; ^2^Research Center for Health Sciences, Hamadan University of Medical Sciences, Hamadan, Iran; ^3^Center of Excellence for Occupational Health, Occupational Health & Safety Research Center, Hamadan University of Medical Sciences, Hamadan, Iran; ^4^Department of Biostatistics and Epidemiology, Modeling of Non-communicable Diseases Research Center, Hamadan University of Medical Sciences, Hamadan, Iran

**Keywords:** Air temperature, Executive functions of brain, Electrocardiogram, Respiratory rate, N-back test

## Abstract

**Background:** This study aimed to investigate the effect size (ES) of air temperature on the executive functions of human brain and body physiological responses.

**Methods:** In this empirical study, the participants included 35 male students who were exposed to 4 air temperature conditions of 18°C, 22°C, 26°C and 30°C in 4 separate sessions in an air conditioning chamber. The participants were simultaneously asked to take part in the N-back test. The accuracy, electrocardiogram (ECG) signals and the respiration rate were recorded to determine the effect of air temperature.

**Results: ** Compared to moderate air temperatures (22°C), high (30°C) and low (18°C) air temperatures had a much more profound effect on changes in heart beat rate, the accuracy of brain executive functions and the response time to stimuli. There were statistically significant differences in the accuracy by different workload levels and various air temperature conditions(P<0.05). Although the heart beat rate index, the ratio between low frequency and high frequency (LF/HF), and the respiratory rate were more profoundly affected by the higher and lower air temperatures than moderate air temperatures (P<0.05), this effect was not statistically significant, which may be due to significant reduction in the standard deviation of normal-to normal intervals (SNND) and the root of mean squared difference between adjacent normal heart beat (N-N) intervals (RMSSD) (P>0.05).

**Conclusion:** The results confirmed that the unfavorable air temperatures may considerably affect the physiological responses and the cognitive functions among indoor employees.Therefore, providing them with thermal comfort may improve their performance within indoor environments.

## Introduction

The office indoor environment quality plays an important role in the health, comfort, welfare and performance of office workers.^1^ This dynamic interaction between workers and the environment, as a key issue in almost all work settings, causes physiological and psychological responses among workers, hence influencing their comfort, efficiency, productivity, safety and health.^2^

The impact of environmental factors on human performance has been studied over the past decades. Researchers first focused more on manual work, while recent studies have mainly focused on the office work which puts a lot of burden on workers’ mental capacity. Various researches have shown that the indoor environment significantly affects human performance. Therefore, modifying the indoor environments is a very important factor in studying the human performance. The indoor environment includes several factors such as temperature, lighting, noise, etc.^3^

Exposure to different air temperatures (temperature variation), the exposure time and differences in participants’ compliance with heat and nutrition are among the factors that may affect human performance.^4^ Therefore, it is highly difficult to assess the relationship between exposure to heat and cognitive function.^4^ Researchers generally agree that very high and very low air temperatures can affect the human performance.^2,5,6^ Other studies have confirmed the impact of air temperature on human performance.^3,6-13^ Obviously, human body warns that there is no heat balance between the body and the environment when it feels hot or cold. Such imbalance can in turn effect human’s health, performance and comfort.^14^

Studies have also shown that, among environmental factors, air temperature and relative humidity have a greater impact on humans because high environmental air temperature results in a great deal of strain on the cardiovascular system to maintain body temperature.^15^

Recent studies have also reported that the increase in air temperature (from 22.8°C to 30.6°C) reduces the performance by 5% due to thermal stress.^16^ Some other studies have shown that performance decreases by 2% due to a 1ºC increase in the air temperature.^17,18^

Some studies have shown that air temperature exercises various effects (e.g. physiological and cognitive effects) in different work environments.^19^ Some studies have demonstrated that air temperature can affect the heart rate variability (HRV).^7,20,21^

The executive functions of the brain can be considered as the cognitive flexibility and the management of intervening elements in goal-oriented behaviors and prediction of the consequences of a function,^22^ which may be affected by exposure to air temperature and negatively affect the cognitive function of the individual.

Valuable information has been published regarding exposure to heat and cognitive function over the past two decades, but there is little empirical evidence for further understanding the issue.^4^ Considering the importance of air temperature as a key environmental factor affecting the indoor environments of offices and industrial control rooms and given that office workers spend 90% of their work time in indoor environments,^1^ the imbalance between temperature level and human task can have the dire consequences, in terms of thermal comfort and performance at work. The difference between this study and other studies is that carrying out the task of working memory and physiological indices are measured at the same time during exposure to different temperatures, which can be reflected in the actual changes in these indices. Therefore, the present study aimed to investigate the effect of indoor air temperature on the executive functions of the brain via the working memory test (at three cognitive levels) and on the physiological responses in the office work station.

## Materials and Methods

### Participants

In this study, sample size was computed by formula down:

$$N=\frac{2{\left(Z_{\alpha }\;+Z_{\beta \;}\right)}^{2\;}(1+(n-1)\rho )}{n[{\left(\mu_{1}\;-\;\mu_{2}\;/\sigma \right]}^{2}}$$

Using similar studies and for α = 5% and β = 95% sample size by rate of abscission 20% was estimated 35 participant male students of Hamadan University of Medical Sciences with age range between 20 to 30 years (mean age 22.96 [SD: 2.96] years) were selected (pre-exposure and post-exposure) as the sample for the study under laboratory conditions. First of all, the subjects were screened for visual and hearing health. The visual test was carried out by E chart.^23^ On the other hand, the hearing health, was conducted by an audiometer (MEVOX ASB15). Upon interviewing the participants, those with a history of neuropsychiatric diseases and the use of drugs that might affect the nervous system were excluded from the study (through interviews with participants). They were also asked to have enough sleep on the night before the test, not to use caffeine and any other stimulant for 12 hours before the study,^24^ and not to smoke. Only male volunteers were selected in this study to control the gender effects. All of the participants were paid for the time they allocated to the study so that they could have further motivation. After the sample members were selected, informed consent for participation in the study was obtained from them and they were assured they could withdraw from the study at any stage without consequences.

### Experimental Procedure

**Figure 1:**
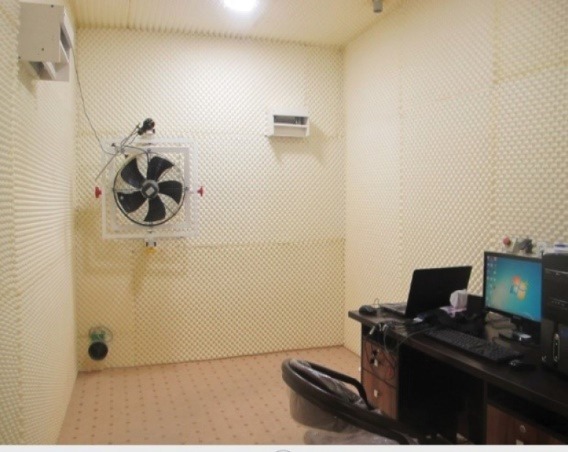
Experimental setup in Air conditioning chamber.

This study was carried out in an air-conditioning chamber with the dimensions of (L × W × H = 3.70 × 2.40 × 2.70 m). The chamber’s walls were covered with brightly colored polyurethane foam (it is an excellent sound absorber), so that it could be psychologically suitable.^25^ The chamber’s floor was covered with cream-colored carpet. There were in this chamber a desk, a chair and a computer which had been ergonomically designed^26^ like the workroom of an office operator (Figure 1), so that the distance from the monitor to the operator was one meter. Moreover, the minimum lighting in the chamber was at least 300 lux,^27^ supplied by two LEDs.

**Figure 2:**
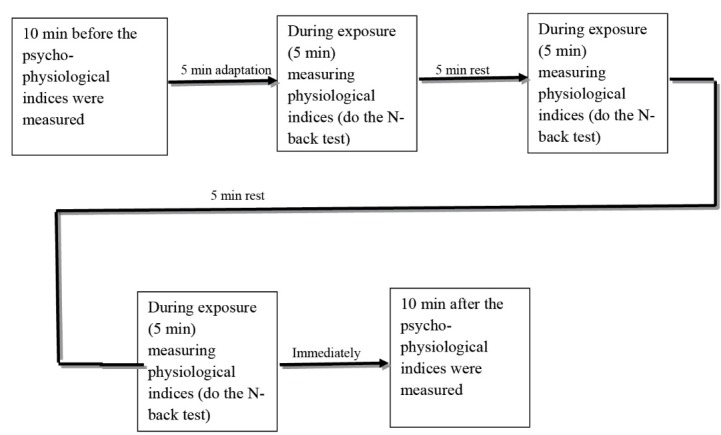
Different stages of measuring physiological indices and mental performance.

The subjects were asked to specify the exact date and time they were ready to conduct the tests, which were performed in four separate sessions. Each person completed all tests in 50 minutes (each day with a constant temperature) and at air temperatures of 18°C, 22°C, 26°C and 30°C. The air temperature of 18 °C is cool and the temperature of 22°C is natural, while 26°C is a little higher than natural. The temperature of 30°C is not unrealistically high for naturally ventilated office environments under summer conditions. The ambient air temperature was checked every ten minutes around the body to resolve any possible difference. The background noise of the chamber was 55 dB (A) and had a low frequency associated with the air-conditioning fans in the chamber (similar to the natural noise in the offices). Before the tests, the participants were trained in two sessions on the test procedure and how to work with the N-back software.^28^ All of the people were working while sitting and the order of doing the job was from low to high workload. The participants in this study were required to wear ordinary clothes such as shirts, pants, underwear, socks and shoes. The air temperature was also under controlled conditions (in different stages of the experiment) with the constant relative humidity of 50% in all conditions and the air flow rate of 0.15 m/s. Each task lasted for 5 minutes and each stimulus was displayed for 2500 ms. There were 120 stimuli at all levels of task executed during exposure to different air temperatures (Figure 2).

When performing each task, the subjects were given a break while sitting on the chair so that they could prepare themselves for the next stage (in order to reduce the effect of fatigue). In order to prevent defects in measurements, the subjects were asked to have a minimum movement and focus on the task. After installation of electrocardiogram (ECG) electrodes and tying the respiration belt around the subjects’ waists, the signals were measured before, during and after the execution of the N-back task for 5 minutes with open eyes.

### Measurements

*Measurement of the Executive Functions of the Brain*

In this study, the N-back test was used to evaluate the executive functions of the brain in three cognitive levels (low workload (n = 1), medium workload (n = 2) and high workload (n = 3). The N-back test is a cognitive function measurement task with executive functions which was first introduced by Kirchner.^29^ In this test, a number of visual stimuli appear on the computer monitoring a random series, and the person should respond in three different conditions with different workload levels. In the low- workload conditions, the person should press the target key in the case of the similarity of each stimulus with the previous stimuli. In the medium workload conditions, the person should compare the stimulus with the two previous stimuli and if similar, press the corresponding key. In the high conditions, the person should compare the stimulus with the three previous stimuli and press the appropriate key if similar. The output of this test contains the number of correct and incorrect answers and the average response time.^28^ In this study, the stimuli were presented as random numbers from 1 to 9 and the number of correct answers (accuracy) and the average response time in milliseconds in the four exposure conditions of air temperature (18°C, 22°C, 26°C and 30°C) were calculated by the N-back software and used at the end of each level of the task. In order to better understand the effects of temperature on the accuracy of participants’ responses in terms of performance with different workloads, the following empirical method was used to study the effects of temperature on accuracy of responses. At first step, the difference was calculated between the temperature of 30°C and the temperature of 22°C. In the second step, the difference between the percentages of correct responses was calculated at 30°C and 22°C. In the third step, the difference between the second step was divided by the difference between the first step.

1. Δt =30°C (is not unrealistically high for naturally ventilated office environments under summer conditions) -22°C (is natural)

2. Accuracy ÷ Δ°C

*Physiological measurements*

To measure the physiological indices, Nexus 4 device (made by Mind Media B.V in the Netherlands) was applied. The Nexus 4 has four channels for measuring the psycho-physiological indicators with Bluetooth technology. The electroencephalography (EEG), ECG, electromyography (EMG) and electrooculogram (EOG) signals can be collected using this system. This system is also able to monitor the skin temperature (TMP); respiratory rate (RSP); galvanic skin resistance (GSR) and blood volume pulse (BVP). The signals are transmitted through wireless networks using Bluetooth to monitor the data online and store them into the computer’s memory. Data processing, digital filtering, reporting of trends and preliminary statistical analysis were performed using the (BioTrace software^®^, Mind Media BV, Roermond - Herten, the Netherlands).^30^ The operational channel information was used to measure ECG signals at 1024 Hz.

ECG signals were recorded by installing three Ag-AgCl electrodes in the right and left sides on the sternum bone and on the sixth rib in the left side of armpit. BioTrance + the use of the ECG signal on the Nexus is the basis of heart rate (HR) and HRV measurement. HRV refers to the beat-beat alterations in HR, which is evaluated based on the ECG in various workload conditions (low, medium and high) and at ambient air temperatures of 18°C, 22°C, 26°C and 30°C. HRV analysis was performed in the following frequency bands: low frequency (LF) (0.04- 0.15 Hz) and high frequency (HF) (0.15-0.4 Hz).^31^ There is evidence that the LF peak is related to sympathetic and parasympathetic activities,^32^ while the HF is related to vagal activity. The LF/HF ratio usually shows the activity of the sympathetic nervous system.^33^ By recording the ECG signals, we measured the mean HR, standard deviation of normal-to-normal intervals (SDNN) in milliseconds, root mean square of successive heartbeat interval differences (RMSSD) in milliseconds and the LF/FH for 5 minutes in four pre-exposure and during-exposure conditions and in three task conditions with low, medium and high workload condition and after exposure to air temperatures of 18°C, 22°C, 26°C and 30°C. In addition, Nexus-4 sensors measure the relative expansion of the abdomen or chest along the inhale and exhale. They are tied as a resilient belt around the body and include small and large sizes. The large size sensor was used in this study in such a way that it was positioned in the abdominal area, so that the sensor was in normal position around the umbilical cord and a layer of shirt was placed between the skin and the sensor for achievement of the best result. The proposed sample rate for the operational channel is used for recording the respiratory rate in the Nexus-4 based on the (NX-RSP1A) 32 SPS sensor. The proposed sample rate (SPS32) of 32 Hz frequency in channel C was used in this study. While the individual was wearing the belt around the abdomen, respiratory signals (RSP) were measured for 5 minutes in the four pre-exposure conditions, in the during-exposure condition in three task conditions with low, medium and high workload levels, and in the post-exposure condition at air temperatures of 18°C, 22°C, 26°C and 30°C.

### Statistical Analysis

The data were analyzed using repeated measure analysis of variance (RM ANOVA).** **Mauchly’s test of sphericity was used for evaluating the natural distribution the data. The post hoc tests were carried out to examine the difference between the means. Significant level for all tests was considered 5% level. The effect size (ES)^34^ was reported. Statistical analysis was used SPSS version 24 (SPSS Inc., Chicago, IL, USA).

## Results

### Results of Measurement of Brain Executive Functions

In Table 1, the mean (SD) of the physical characteristics of the age (y), height (cm), weight (kg) and body mass index (kg/m^2^) of the participants are presented. The results of the executive functions of the brain including the mean values, standard deviation, *P* value and ES for the three conditions with different workload levels and the four air temperature conditions based on average accuracy in percentage and average response speed (mean response time to stimuli) in milliseconds (ms) are presented in Table 2.

As observed in Table 2, the ANOVA test with repeated measures showed that there was a significant difference between the mean accuracy in task conditions with low workload, medium workload, high workload and air temperatures of 18°C, 22°C, 26°C, and 30°C (*P*<0.05).

The post-hoc tests showed that air temperature had a significant effect on the mean accuracy at air temperatures of 18°C, 26°C and 30°C compared to the air temperature of 22°C in task conditions with medium workload and high workload. As a result, there was a significant difference between the mean accuracy in task conditions with low workload (n=1) and air temperatures of 18°C and 30°C as well as 22°C and 30°C (*P* <0.05). Also, significant differences were detected between the mean accuracy in task conditions with medium workload (n=2) and air temperatures of 18°C and 22°C, 22°C and 26°C, 22°C and 30°C, 26°C and 30°C (*P* <0.05). Further, there was a significant difference between the mean accuracy in task conditions with high load (n=3) and the air temperatures of 18°C and 22°C as well as 22°C and 30°C (*P* <0.05).

In addition, as shown in Table 2, the highest mean of response to stimuli was related to the air temperature of 22°C in all conditions, whereas the lowest response rates to the stimuli in task conditions with low workload (n = 1) and medium workload (n = 2) in task conditions with low workload (n = 1) and medium workload (n = 2) were registered in the air temperature of 18°C. It was also associated with the air temperature of 30°C in task conditions with high workload (n = 3).

Table 3 displays that increasing and decreasing ambient temperature relative to comfort temperature (22°C) affected the response accuracy of the participants. Based on equations (section measurement of the executive functions of the brain) show that with an increase of 1°C of ambient air temperature, the accuracy of responses while performing low workload, medium workload and high workload reduced by 1.39%,1.45% and1.31% respectively. If the same calculations were made for a decrease of 1°C relative to the comfort temperature (22°C), we would conclude that by 1°C reduction of ambient temperature, the response accuracy in low workload, medium workload and the high workload would drop by 0.54%, 1.97%, and 2.57% respectively.

We also evaluated the ES 18°C, 26°C and 30°C than 22°C on the mean accuracy, the results of which are summarized in Table 3.

### Results of measurement of physiological indices

Table 4 demonstrates physiological indices for different conditions of measurement in comparison with the baseline (pre-exposure) condition. The results showed the increased difference between the pre-exposure and post-exposure mean changes at the air temperatures of 18°C, 22°C, 26°C and 30°C for HR, LF/HF and RSP indices in different conditions of measurement and a decreased difference for the SDNN and RMSSD indices except for the air temperature of 22°C.

As indicated in Table 4, the results of the ANOVA test with repeated measures showed that the air temperature had an effect on HR, which was statistically significant between the pre- and post-exposure levels (*P*<0.05). The post-hoc test showed that there was a significant difference between the HR at the air temperatures of 18°C and 26°C, 18°C and 30°C, 22°C and 26°C, and 22°C and 30°C (*P* < 0.05).

The results of the analysis of variance test with repeated measures showed significant differences in the mean HR of the participants in task conditions with low workload, medium workload and high workload in comparison with the baseline condition in the air temperatures of 18°C, 22°C, 26°C and 30°C (*P* < 0.05). The results of the post-hoc test also indicated a significant difference between the increased changes in the mean HR in task conditions with low workload and the air temperatures of 18°C and 26°C, 18°C and 30°C, 22°C and 26°C, and 22°C and 30°C (*P* < 0.05).

The results of the post - hoc test revealed that there was a significant difference between the mean HRV as compared to the baseline in task conditions with medium workload and air temperatures of 18°C and 30°C, 22°C and 26°C, and 22°C and 30°C (*P* < 0.05).

Moreover, the post hoc test results showed a significant difference between the increase of the mean HR variability in comparison with the baseline condition in the task conditions with high workload and air temperatures of 18°C and 30°C, 22°C and 26°C, and 22°C and 30°C (*P* < 0.05).

The analysis of variance test with repeated measures showed no significant difference between the variations in the mean values of SDNN in pre- and post-exposure conditions in different air temperatures of 18°C, 22°C, 26°C and 30°C (*P* > 0.05). Moreover, the results of the ANOVA test with repeated measures did not show a significant difference between the increase in the mean SDNN variability in task conditions with low workload, medium workload and high workload in comparison with the baseline and in air temperatures of 18°C, 22°C, 26°C, and 30°C (*P* > 0.05).

The analysis of variance test with repeated measures showed no significant difference between variability in the mean values of RMSSD in pre- and post-exposure conditions in ambient air temperature of 18°C, 22°C, 26°C and 30°C (*P* > 0.05). Moreover, the analysis of variance test with repeated measures did not show a significant difference between the mean values of RMSSD in task conditions with low workload, medium workload, and high workload in comparison with the baseline considering the air temperatures of 18°C, 22°C, 26°C, and 30°C (*P* > 0.05).

The results of the analysis of variance test with repeated measures showed that there was a significant difference between changes in the mean values of LF/HF in the pre- and post-exposure conditions in air temperatures of 18°C, 22°C, 26°C and 30°C (*P* < 0.05). However, the analysis of variance test with repeated measures did not show a significant difference between the mean values of LF/HF in task conditions with low workload, medium workload and high workload as compared to the baseline and air temperatures of 18°C, 22°C, 26°C and 30°C, despite a significant increase in air temperatures of 18°C and 30°C.

The analysis of variance test with repeated measures showed that air temperature influenced the respiration rate, and there was a significant difference between changes in the mean values of RSP in the pre- and post-exposure conditions in temperatures of 18°C, 22°C, 26°C and 30°C (*P* < 0.05). In addition, the analysis of variance test with repeated measures indicated a significant difference between the mean RSP changes in task conditions with low workload, medium workload and high workload and the air temperatures of 18°C, 22°C, 26°C and 30°C (*P* < 0.05).

The results of the post hoc test showed a significant difference between the mean values of RSP in task conditions with low workload and air temperature ranging between 22°C and 30°C (*P* <0.05). Moreover, the post-hoc test indicated a significant difference between the variability of the mean values of RSP in task conditions with medium workload and the air temperatures of 22°C and 30°C, 26°C and 30°C (*P* <0.05). The results of the post-hoc test also demonstrated that there was a significant difference between the changes in the mean values of RSP in task conditions with high workload and the air temperatures of 22°C and 30°C, 26°C and 30°C (*P* <0.05).

We also evaluated the effect of the task levels in the air temperature conditions of 18°C, 22°C, 26°C and 30°C, the results of which are summarized in Table 5. Table 5 presents the mean values and the standard errors of the changes of physiological indices and the results of the ANOVA test for task levels with high, medium and low workload levels, and four air temperature conditions. The results showed that the task levels and air temperature conditions could not interact on HR, SDNN, RMSSD, LF/HF and RSP variables.

## Discussion

The results of this study showed that air temperature affects the executive functions of the brain and the physiological indices. The study also suggests that participants had better performance (accuracy) in 22°C compared to 30°C and 18°C. In other words, the air temperature was more pleasant in the neutral air temperature range (22°C) and the participants had a good thermal comfort at these air temperatures, so their performance was less affected by the air temperature.

The results of previous studies have shown that performance is less affected at neutral to slightly warm temperatures,^35‚36^ which is consistent with the results of this study. Studies have also revealed that hot environments are more risky than cold environments; therefore, it is recommended that the ambient air temperature conditions range should before slightly cold to neutral air temperatures,^3^ which is consistent with the results of the study. Seppänen and colleagues’^6^ studies on the work in various air temperature showed that the highest efficiency was associated with neutral air temperature, which is consistent with the results of this study. Many studies have shown that an increase of 1°C leads to a decrease in performance by 2%, and no change in performance has been observed in the moderate air temperature range. This has also been corroborated by Tanabe et al.^18^

The results of this study showed that with an increase of 1°C ambient temperature compared to comfort temperature (22°C), the accuracy of response performance in terms of low workload, medium workload and high workload declined by 1.39%, 1.45% and 1.31% respectively. On the other hand, the results of this study showed that with a reduction of 1°C in ambient temperature compared to comfort temperature (22°C), the accuracy of responses performance in terms of low workload, medium workload and high workload declined by 0.54%, 1.97% and 2.57% respectively. These results indicate that workplace authorities should concentrate on employee safety, as well as the efficiency and quality of their work, especially in control rooms, offices and other similar environments.

The results of this study were not consistent with the findings of Fang et al^36^ and Kahl.^37^ Perhaps this difference is caused by laboratory conditions, gender or even the kind of task that participants performed while being exposed to various temperature. Another main reason for this contradiction may be the participants’ gender. In our study, all participants were male, while in Fang et al study, all of them were female, and in Kahl’s research, 140 of the participants were female and only 36 were male. The results showed that, on average, subjects decreased their response times in the low and high temperature (18°C and 30°C) compared the other temperature conditions. A shorter response time corresponds to a greater stress from the subject, according to the arousal theory.^37^

On the other hand, the human body reacts to environmental stimuli and there is a continuous and dynamic interaction between the human body and the environment, which creates a psychological and physiological strain.^2^ A study conducted by Yao et al^20^ showed that HRV has a close relationship with thermal comfort. Our study also displayed that the air temperature affects the mean of HR indicators, LF/HF, SDNN, RMMSD and respiration rates in performance conditions at different levels. As a result, there was a significant difference between the mean HR changes, with the highest level of the obtained significance being observed in air temperatures of 30°C and 22°C at all levels of task execution. Liu et al^21^ concluded that the HR changes at high air temperatures was higher than that in moderate air temperatures, which is consistent with the findings of this study.

This study also showed that there were greater changes in LF/HR ratio of HRs when participants were exposed to undesirable air temperatures (high and low air temperatures) than when they were exposed to moderate air temperatures. This may be due to heat feeling discomfort at these air temperatures. This finding is consistent with the results of previous studies.^7,20,38^ The present study also showed that the changes in the mean SDNN and RMSSD in exposure to unsuitable conditions (30°C and18°C) were greater than those in the exposure to 22°C. In this study, the changes in the mean RSP were higher in 30°C than in 22°C, so that there was a significant difference between the changes in the RSP in 30°C as compared to the changes in 22°C, which is consistent with the results of the study conducted by Liu et al.^21^ In general, changes observed in physiological parameters with increasing and decreasing air temperature relative to the thermal comfort temperature (22°C) indicate that warm temperatures and cold temperatures have imposed stress on the participants.

### Advantages and limitations of the present study

In general, one of the strengths of this study was measuring the physiological indices simultaneously in five pre-exposure (base line), and during-exposure conditions with low, medium and high workload levels in various air temperatures immediately after exposure, which could record the instantaneous changes in the physiological characteristics of HRV and respiration rates (RSP) in different conditions. This condition can provide better judgment on the effect of air temperature on the executive functions of the brain, and the physiological indices of participants. However, the results of this study provide useful evidence on the effect of unfavorable air temperatures on the physiological and mental assessments, which can be effective in improving the interaction of environmental and cognitive ergonomics for creating the air temperature conditions for the indoor environments of offices.

Limitations of this study that need to be addressed here include the following. All of the participants in this study were males and aged 20-30 years and were not very similar to those in the workplace. On the other hand, participants in this type of study seem to demonstrate their best performance, which can cover the adverse effects of environmental factors on cognitive function. Therefore, it seems that it cannot show the true scenario of being in the real environment. Also, the impact of gender has been disregarded in this study. It is advisable to use office workers in real work environments in the future research.

## Conclusion

It can be concluded from the laboratory assessment of the effect of air temperature on the executive functions of the brain and the physiological parameters that:

- Brain executive functions in high and low air temperatures are more influenced by the air temperature than the moderate air temperature, which may reduce accuracy and increase the error in sensitive work environments that required more attention.
- High and low air temperatures significantly increased the participants’ HR, LF/HF and respiratory rates, which can have a represents stress and high mental fatigue and negative long-term impact on their health.
- Undesirable high and low air temperatures caused significant changes in the SDNN and RMSSD indices, which have not been uniformly changed at different air temperatures.
- In moderate air temperatures (22°C), the LF/HF ratio, which represents the sympathetic and parasympathetic equilibrium to the vagal one, is approximately close to one, indicating that, in this temperature, the participants had a better thermal comfort, so that they had a good performance (accuracy).

## Ethical approval

This study was approved by the Research Ethics Committee of Hamadan University of Medical Sciences (grant No. 9510146069). All participants signed the consent form and they were ensured of the confidentiality of data collection prior to the study.

## Competing interests

The authors declare that they have no competing interests.

## Authors’ contributions

MM and MA conceptualized the study, designed data collection methodology, and led manuscript development. AMA collected all the data and wrote the first draft of the manuscript. RG contributed to the writing of the manuscript. LT conducted data analyses.

## Acknowledgments

We greatly appreciate the students of Hamadan University of Medical Sciences for participation in this project. This paper has been extracted from a Ph.D. dissertation at Hamadan University of Medical Sciences.

**Table 1:** Demographic characteristics of the participants

**Specifications**	**Mean± SD**
Age (y)	22.96±2.96
Height (cm)	178.08±4.93
Weight (kg)	72.91±10.33
BMI (kg/m^2^)	22.98±2.98

**Table 2:** Means ± SD of accuracy and response speed (average response time) of working memory test in three levels with different workload under different ambient air temperatures

**Parameter**	**18°C**	**22°C**	**26°C**	**30°C**	***P*** ** value**	**ES**
Accuracy n=1(%)	87.05±9.93	89.24±12.22	84.22±12.22	78.11±13.86	<0.000	0.151
Accuracy n=2(%)	77.80±11.57	85.69±6.36	80.20±11.00	74.02±12.97	<0.000	0.185
Accuracy n=2(%)	73.22±12.73	82.52±7.12	77.88±11.80	71.99±12.04	<0.001	0.155
Response time (ms), n=1	430.17±160.29	449.49±130.04	470.54±114.04	459.183±136.41	<0.636	0.016
Response time (ms), n=2	485.02±150.06	530.97±1530.79	525.20±160.27	506.63±135.26	<0.437	0.026
Response time (ms), n=3	523.29±159.89	575.60±156.35	569.71±83.02	563.89±136.60	<0.297	0.036

Accuracy: Percentage of correct answers. Response time: Average response rate to the stimuli n=1, n =2 and n=3, respectively: low workload, medium workload, high workload‚ ES: Effect size.

**Table 3:** Pair wise comparison of the effect of various temperatures on the accuracy

**Comparison of air temperatures**	**Accuracy (%)**		
	**MD±SE**	**ES**	***P***
18°C vs. 22°C, n=1	2.19±2.27	0.026	0.345
26°C vs. 22°C, n=1	5.02±2.48	0.107	0.051
30°C vs. 22°C, n=1	11.12±2.85	0.308	0.000
18°C vs. 22°C, n=2	7.89±2.14	0.285	0.001
26°C vs. 22°C, n=2	5.49±2.04	0.175	0.011
30°C vs. 22°C, n=2	11.66±2.50	0.390	0.000
18°C vs. 22°C, n=3	9.30±2.29	0.326	0.000
26°C vs. 22°C, n=3	4.64±2.67	0.081	0.092
30°C vs. 22°C, n=3	10.55±2.32	0.379	0.000

^a^ *P* < 0.05 MD: Mean of difference‚ n = 1, n = 2 and n = 3, respectively: low workload, medium workload, high workload, ES: effect size, SE: Standard error.

**Table 4:** Means ± SE of changes in heart rate variation indexes and respiration rates in the conditions of performing three levels of tasks and four different air temperature conditions

**Variables (Mean difference)**	**18°C**	**22°C**	**26°C**	**30°C**	***P*** ** value**
HR (beat/min)	0.43±0.951	1.37±0.498	7.84±0.980	10.98±1.72	<0.000
HR (beat/min), n=1	-1.11±1.07	-2.42±1.34	1.96±0.778	2.71±1.08	<0.038
HR (beat/min), n=2	2.18±0.923	-0.16±0.996	5.13±1.38	5.91±1.23	<0.001
HR (beat/min), n=3	2.44±0.885	2.10±0.508	5.51±1.50	7.35 ±1.32	<0.003
SDNN (ms)	-22.39±9.30	2.59±5.79	-14.89±14.02	-16.57±8.35	<0.624
SDNN (ms), n=1	-17.13±10.56	-0.34±14.25	-5.4±8.43	-8.66±7.87	<0.557
SDNN (ms), n=2	-19.18±13.58	-8.37±8.93	-17.53±12.53	-19.85±8.04	<0.758
SDNN (ms), n=3	-23.30±14.25	-12.33±7.79	-18.34±12.73	-21.81±8.68	<0.856
RMSSD (ms)	-11.80±16.12	8.53±7.71	-10.26±18.87	-21.47±7.31	<0.457
RMSSD (ms), n=1	-13.56±13.34	-0.23±7.30	-6.85±18.79	-9.05±6.55	<0.840
RMSSD (ms), n=2	-19.91±15.21	-7.80±10.66	-11.89±19.00	-13.91±7.14	<0.902
RMSSD (ms), n=3	-20.93±12.10	-8.26±10.55	-13.86±19.46	-16.42±7.15	<0.872
LF/HF (%)	2.29±1.02	1.03±1.25	3.40±0.576	7.16±1.20	<0.000
LF/HF (%), n=1	2.04±1.17	0.43±1.19	1.35±0.558	1.57±0.521	<0.567
LF/HF (%), n=2	2.78±1.15	0.54±1.04	1.84±0.680	2.29±0.589	<0.297
LF/HF (%), n=3	5.28±1.07	0.93 ±1.13	2.39±0.537	6.63± 4.55	<0.298
RSP (bpm)	1.72±0.567	0.11±0.803	2.50±0.452	4.55±0.561	<0.000
RSP (bpm), n=1	2.24±0.557	1.01±0.760	2.40±0.495	3.44±0.483	<0.049
RSP (bpm), n=2	2.51±0.727	1.12±0.855	2.70±0.499	4.03±0.527	<0.045
RSP (bpm), n=3	3.57±0.679	2.05±0.807	3.54±0.574	4.88±0.365	<0.038

HR: Heart rate changes as compared with the baseline‚ SDNN: Changes standard deviation of normal-to-normal intervals as compared with the baseline‚ RMSSD: Changes root of the mean squared difference between adjacent normal heart beat (N-N) intervals as compared with the baseline‚ LF/HF: Change in LF to HF ratio as compared with the baseline‚ RSP: change in respiratory as compared with the baseline ‚ n=1, n = 2 and n = 3, respectively, low workload‚ medium workload, and high workload.

**Table 5:** Comparison of means ± SE of changes in heart rate variation indexes and respiration rates in three levels of tasks under different ambient air temperatures

**Variables( Mean differences)**	**18°C**	**22°C**	**26°C**	**30°C**	**Repeated measure ANOVA result**						
					**Heat P-value**	**Task P-value**	**Heat•Task P-value**	**Heat** **ƒ**	**Task** **ƒ**	**Heat•TasK** **ƒ**	**ε**
HR, n=1 (beat/min)	-1.11±1.07	-2.41±1.34	1.96±0.778	2.71±1.08	‏**	‏**	0.835	0.169	0.421	0.012	0.781
HR, n=2 (beat/min)	2.18±0.923	-0.16±0.996	5.13±1.38	5.91±1.23							
HR, n=3 (beat/min)	2.44±0.885	2.10±0.508	5.51±1.50	7.35±1.32							
SDNN, n=1 (ms)	-17.13±10.56	-0.34±14.25	-5.43±8.43	-8.66±7.87	0.80		0.917	0.010	0.114	0.005	0.461
SDNN, n=2 (ms	-19.18±13.58	-8.37±8.93	-17.53±12.53	-19.85±8.04							
SDNN, n=3 (ms)	-23.30±14.25	-12.33±47.79	-18.34±12.73	-21.81±8.68							
RMMSN, n=1 (ms)	-13.56±13.24	-0.23±7.30	-6.85±10.79	-9.05±6.55	0.816	0.147	0.998	0.006	0.055	0.001	0.491
RMMSD, n=2 (ms)	-19.91±15.21	-7.80±10.66	-11.89±19.00	-13.91±7.14							
RMMSD, n=3 (ms)	-20.93±12.10	-8.26±10.55	-13.86±19.46	-16.43±7.15							
LF/HF ratio, n=1	2.04±1.17	0.43±1.19	1.35±0.558	1.57±0.521	‏**	0.051	0.428	0.481	0.10	0.019	0.241
LF/HF ratio, n=2	2.78±1.15	054±1.04	1.84±0.680	2.29±0.528							
LF/HF ratio, n=3	5.28±1.07	0.93±1.13	2.39±0.537	6.63±4.55							
RSP, n=1 (bpm)	2.24±0.557	1.01±0.760	2.40±0.495	3.44±0.843	‏**	‏**	0.995	0.105	0.277	0.003	0.906
RSP, n=2 (bpm)	2.51±0.727	1.12±0.855	2.70±0.499	4.04±0.527							
RSP, n=3 (bpm)	3.57±0.679	2.05±0.807	3.54±0.574	4.88±0.365							

HR: Heart rate changes as compared with the baseline‚ SDNN: Changes standard deviation of normal-to-normal intervals as compared with the baseline‚ RMSSD: Changes root of the mean squared difference between adjacent normal heart beat (N-N) intervals as compared with the baseline‚ LF/HF: LF/HF: Change in LF to HF ratio as compared with the baseline‚ RSP: change in respiratory as compared with the baseline‚ n=1, n = 2 and n = 3, respectively, low workload‚ medium workload, and high workload.
